# Supplementary material for: Knowledge and awareness of bovine tuberculosis associated with raw milk and under-cooked meat contamination among cattle farmers in selected parts of zambia
Source: PLoS Negl Trop Dis. 2025 Apr 10;19(4):e0012870. doi: 10.1371/journal.pntd.0012870 (PMC12017525; doi:10.1371/journal.pntd.0012870)
Supplement: Attachment — Submitted filename: Review Comments.pdf [file pntd.0012870.s002.pdf]

Review Comments

The following issue should be revised by the Author to make the manuscript publishable. The comments are as follow:

Abstract:
Overall, the abstract provides a clear overview of the study's key finding, with practical implications for public health in Zambia.

Introduction:

The background on TB and bTB is informative but could benefit from a more focused introduction to the study's specific objectives. Explicitly mention how the study contributes to existing research knowledge gaps in Zambia.

Methodology:

1. The rationale for choosing Lundazi and Monze districts is well-explained, highlighting the relevance of high cattle population and human-animal interaction. However, consider providing more context on the selection of villages within the districts, for example were they also based on any specific disease burden or other criteria?
2. The methodology on data collection is thorough. A minor suggestion would be to clarify how the respondents were selected. Was it purely random sampling within villages, or is there any stratification occur (e. g., by occupation or exposure risk)?
3. More information on the practical aspects of data collection, such as the duration of interviews or any challenges faced during fieldwork, would add depth to the methodology section.

Results:
The Quantitative results: The sociodemographic data is clear. It would be helpful to present a brief explanation of why a significant proportion of males (86.79%) participated, as this could indicate gender-related patterns in this study area.

The Qualitative results: The four key themes are well-defined, and the qualitative data offers valuable insight into local knowledge and practices. However, the richness of qualitative data could be better conveyed if more illustrative quotes were presented, especially to show variations across different subgroup (e.g., farmers vs veterinarians)

Discussion and conclusion:

1. in some places, there are contradiction between your findings and those from other studies. For instance, the comparison between your study and Yusul's findings regarding age and education levels could benefit from further elaboration on why such discrepancies might exist.

2. There is no mention of potential limitations or gaps in the data, such as the absence of data from woman (due to cultural reasons or the study design) or the fact that your study was conducted only in two districts. Moreover, limitations on the sample size or methodological constraints should be acknowledged in the discussion.

Final comments:
Overall, the study provides valuable insight into bTB awareness in Zambia and offers important recommendations for improving knowledge and prevention. The findings are important for understanding zoonotic disease transmission, and the practical recommendations have the potential to make real impact.

Considerations:
ACCEPTED WITH MINOR CORRECTION

Reason to accept:
1. The study provides valuable insight into cattle farmers awareness of the transmission of bTB, focusing on cultural and social practice that pose risks that highly relevant to public health and agricultural sectors, especially in developing country.
2. The article contributes to a deeper understanding of how demographic, educational, and occupational factors affect knowledge about bTb.
