# Supplementary material for: Knowledge and awareness of bovine tuberculosis associated with raw milk and under-cooked meat contamination among cattle farmers in selected parts of zambia
Source: PLoS Negl Trop Dis. 2025 Apr 10;19(4):e0012870. doi: 10.1371/journal.pntd.0012870 (PMC12017525; doi:10.1371/journal.pntd.0012870)
Supplement: Attachment — Submitted filename: A REBUTTAL LETTER.docx [file pntd.0012870.s004.docx]

A REBUTTAL LETTER

METHODS

Reviewer #1:

1. The objectives of the study are somewhat implied, but there isn’t a clearly stated, testable hypothesis in the typical format. The aim is to assess the level of awareness regarding bovine tuberculosis and identify the factors contributing to its transmission, but a more specific, testable hypothesis would strengthen the research design. For example: "Cattle farmers have lower awareness of bTB transmission compared to veterinary professionals."

Action.

Null Hypothesis(H0): There is no significant difference in the level of awareness about the risks associated with bTB transmission through raw milk and undercooked meat contamination between cattle farmers and veterinarians

Alternative Hypothesis (H1): Cattle farmers will have a lower level of awareness about the risks associated with bTB transmission through raw milk and undercooked meat contamination compared to veterinarians

2. Yes, the mixed-methods design (quantitative through surveys and qualitative through focus groups) is appropriate for addressing the study’s objectives. The quantitative approach allows for measuring awareness levels across different groups, while the qualitative approach provides in-depth insights into the cultural and social practices that may contribute to bTB transmission.

Affirmative

3. Yes, the population is clearly described. The study includes respondents from two districts in Zambia (Lundazi and Monze) with cattle farmers, abattoir workers, veterinary professionals, and commercial businessmen. The focus on these groups is appropriate for understanding the awareness of bTB transmission and the factors contributing to its spread, as they are directly involved with cattle handling and consumption of animal products.

Affirmative

4. The sample size appears to be adequate for a descriptive cross-sectional study. A total of 280 respondents, including both quantitative survey participants and qualitative key informants, is a reasonable number for drawing conclusions on awareness levels. However, the power of the study would depend on the statistical analysis and the size of the subgroups being compared (e.g., cattle farmers vs. veterinary professionals). The sample size for the qualitative aspects (focus groups and key informants) is smaller, but this is typical for qualitative data.

Affirmative

5. Yes, appropriate statistical methods were used. Descriptive statistics were used to summarize the data, and multinomial logistic regression was applied to assess the relationships between factors such as meat consumption, milk consumption, and handling. The use of R software for analysis is standard and appropriate for handling the quantitative data. For qualitative data, Nvivo was used for coding and identifying themes, which is a valid method for qualitative analysis.

Affirmative

6. Yes, ethical considerations were appropriately addressed. The study received approval from the University of Zambia's Biomedical Research Ethics Committee, and participants were informed about the study's nature and their rights, including the right to privacy, voluntary participation, and informed consent. These ethical measures seem to be in line with common standards for human research.

**RESULTS**

Reviewer #1:

1. Yes, the analysis presented matches the analysis plan outlined in the methodology. Quantitative data was analyzed using descriptive statistics, and multinomial logistic regression, which is consistent with the described approach. The qualitative data was analyzed by coding the focus group discussions and interviews into major themes and sub-themes using Nvivo, which aligns with the planned qualitative analysis approach. The results from both quantitative and qualitative analyses are clearly presented in the study.

Affirmative

2. Yes, the results are presented clearly, but there could be some improvements in terms of completeness and detail. For example:
   The quantitative results are summarized in terms of statistical significance (p-value < 0.001) and the odds ratio (OR = 3.12), which provide an understanding of the difference in knowledge between various groups (such as cattle farmers versus veterinary professionals).
   The qualitative findings are organized by themes (knowledge of bTB, unpasteurized milk and uncooked meat consumption, and handling beef with bare hands). Specific quotes from participants are included to illustrate these themes, which strengthens the depth of the findings. However, a more detailed presentation of the results, such as how the statistical tests (e.g., multinomial logistic regression) were used to assess the relationship between knowledge and behaviors, would enhance the clarity of the results. Including specific data tables (like Table 1) and providing the exact significance levels or confidence intervals would offer more comprehensive insight.

Veterinarians with excellent knowledge and awareness of bTB were more likely to practice excellent knowledge and behavior towards Bovine prevention and control (OR=10.5, 95% CI=5.5-22.3).

3. Yes, Figures and Tables provide sufficient detail for the reader to understand the results easily.

Affirmative

**Review Comments**

**Abstract:**

Overall, the abstract provides a clear overview of the study’s key finding, with practical implications for public health in Zambia.

Affirmative

**Introduction**

The background on TB and bTB is informative but could benefit from a more focused introduction to the study’s specific objectives. Explicitly mention how the study contributes existing research knowledge gaps in Zambia.

The study significantly contributes to the management practices of Bovine Tuberculosis (bTB) in line with the main motivation in the fields of animal and human health, which is to enhance the understanding of the effectiveness of the current government regulatory framework on bTB in Zambia. Further,the study findings fills a knowledge gap on the level of awareness of bTB among cattle farmers and veterinary professionals in Zambia. Previous a lot of studies have focused on prevalence of bTB in Zambia, however very few have examined awareness levels of bTB among stakeholders

**Methodology**

1. The rationale for choosing Lundazi and Monze districts is well-explained, highlighting the relevance of high cattle population and human-animal interaction. However, consider providing more context on the selection of villages within the districts, for example were they also based on any specific disease burden or other criteria?

The elected villages where located close to veterinarian camps , which makes the researcher to access other relevant information pertaining the disease ,additionally villages were accessible by roads, which made it easier for the research team to conduct the study more especially, in that in Lundazi the studies were conducted during the rain season. Hence some roads where impassable

2. The methodology on data collection is thorough. A minor suggestion would be to clarify how the respondents were selected. Was it purely random sampling within villages, or is there any stratification on occur (e. g., by occupation or exposure risk)?

Purposive sampling method was used to select respondents within the villages

3. More information on the practical aspects of data collection, such as the duration of interviews or any challenges faced during fieldwork, would add depth to the methodology section

All interviews lasted close to 60 minutes, which was well within the recommended 90 minutes for an interview (Seidman, 2006). Two methods were used to record the data. To begin, the researcher acted as both a recorder and an interviewer by keeping track of the responses in a field notebook. Second, with the permission and consent of the interviewees, the researcher used a voice recorder to record the interviews. Data gathering was a big challenge .Additionally, due to time and resource constraints, the study could not cover all the cattle farmers in Zambia but focused mainly on cattle farmers in the selected areas of Eastern and Southern Provinces. Not all targeted cattle farmers were captured in the Eastern Province due to the fact that during the researcher’s visit it was rainy season, many roads were impassable and a number of cattle farmers were busy tilling their land

**Results:**

The Quantitative results: The Socio-demographic data is clear. It would be helpful to present a brief explanation of why a significant proportion of males (86.79%) participated this could indicate gender-related pattern in this study area.

Our findings revealed more males than females in cattle farming because cattle farming and operations often requiring physically demanding work. In addition, Zambian cattle farming, men are expected to take on more responsibilities pertaining to livestock farming, while women are directly involved in household management

The Qualitative results: The four key themes are well-defined, and the qualitative data offers valuable insight into local knowledge and practices. However, the richness of qualitative data could be better conveyed if more illustrative quotes were presented, especially to show variation across different subgroup (e.g., farmers vs veterinarians)

*Unpsterised milk and undercooked meat are significant risk factors for bTB transmission. There is need to advise cattle farmers and the general public about these risks."*

*(Veterinarian officer, Lundazi)*

*"Bovine tuberculosis can be transmitted from animals to humans. I have observed that cattle farmers have been consuming raw milk and undercooked meat, therefore we need to take this seriously and educate the farmers and public about the risks."*

*(Humam doctor; Monze)*

*"We need better training on how to handle meat safely and prevent the spread of diseases like bTB."*

*(Male abattoir worker, Monze)*

**Discussion and conclusion:**

1. In some places, there are contraction between your findings and those from other studies. For instance, the comparison between your study and Yusul’s findings regarding age and education levels could benefit from further elaboration on why such discrepancies might exist.

Age

Youthful respondents in the study area may prioritize very immediate needs, such as clothes, food and shelter, over long-term investments in bTB prevention and control.. hey may have limited exposure to cattle farming and bTB,

Respondents aged 50 years and above may not have had regular interactions with veterinary professionals, which could limit their opportunities to learn about bTB. Elderly respondents may have experience memory decline, which could affect their ability to learn and retain new information.

Education

Those respondents with low education levels may be more likely to live in absolute poverty, which can limit access to information about Bovine tuberculosis.They may as well may hold traditional beliefs and practices that do not prioritize bTB prevention and control

2. There is no mention of potential limitation or gaps in the data, such as the absence of data from woman (due to cultural reasons or the study design) or the fact that your study was conducted only in two districts. Moreover, limitations on the sample size or methodological constraints should be acknowledged in the discussion.

The findings from a sample size of 208 may not be representative of the larger population, which can limit the generalization of the finding,Women are very much involve in the household management, therefore cultural limitations may have prevented women from participating in the study, which can limit the understanding of their perspectives and experiences.Further the study was only conducted in two districts, which may not be representative of the entire nation.The study may have been subject to measurement and systematic errors, such self reporting, social desirability bias or recall bias. However, the study attempted to ensure internal validity and reliability by triangulation of quantitative and qualitative findings. Lastly, the study was not designed to estimate the prevalence of Bovine tuberculosis, therefore, further study to conduct a nationwide study to determine the prevalence and incidence of Bovine tuberculosis in Zambia should be undertaken.
