# Supplementary material for: Knowledge and awareness of bovine tuberculosis associated with raw milk and under-cooked meat contamination among cattle farmers in selected parts of zambia
Source: PLoS Negl Trop Dis. 2025 Apr 10;19(4):e0012870. doi: 10.1371/journal.pntd.0012870 (PMC12017525; doi:10.1371/journal.pntd.0012870)
Supplement: Attachment — Submitted filename: A rebuttal letter 14 March.docx [file pntd.0012870.s005.docx]

**Reviewers' comments:**

**Figure resubmission:**

While revising your submission, please upload your figure files to the Preflight Analysis and Conversion Engine (PACE) digital diagnostic tool, <https://pacev2.apexcovantage.com/>. PACE helps ensure that figures meet PLOS requirements. To use PACE, you must first register as a user. Registration is free. Then, login and navigate to the UPLOAD tab, where you will find detailed instructions on how to use the tool. If you encounter any issues or have any questions when using PACE, please email PLOS at [figures@plos.org](mailto:figures@plos.org). Please note that Supporting Information files do not need this step. If there are other versions of figure files still present in your submission file inventory at resubmission, please replace them with the PACE-processed versions.

DONE

**Reproducibility:**

To enhance the reproducibility of your results, we recommend that authors of applicable studies deposit laboratory protocols in [protocols.io](http://protocols.io/), where a protocol can be assigned its own identifier (DOI) such that it can be cited independently in the future. Additionally, PLOS ONE offers an option to publish peer-reviewed clinical study protocols. Read more information on sharing protocols at <https://plos.org/protocols?utm_medium=editorial-email&utm_source=authorletters&utm_campaign=protocols>

https://orcid.org/0009-0001-5604-8455

DONE
